# Projected future climate change and Baltic Sea ecosystem management

**DOI:** 10.1007/s13280-015-0654-8

**Published:** 2015-05-28

**Authors:** Agneta Andersson, H. E. Markus Meier, Matyas Ripszam, Owen Rowe, Johan Wikner, Peter Haglund, Kari Eilola, Catherine Legrand, Daniela Figueroa, Joanna Paczkowska, Elin Lindehoff, Mats Tysklind, Ragnar Elmgren

**Affiliations:** Department of Ecology and Environmental Science, Umeå University, 901 87 Umeå, Sweden; Swedish Meteorological and Hydrological Institute, 426 71 Västra Frölunda, Sweden; Department of Ecology, Environment and Plant Sciences, Stockholm University, 106 91 Stockholm, Sweden; Department of Chemistry, Umeå University, 901 87 Umeå, Sweden; Centre for Ecology and Evolution in Microbial model Systems - EEMiS, Linnaeus University, 391 82 Kalmar, Sweden; Swedish Meteorological and Hydrological Institute, 601 76 Norrköping, Sweden; Umeå Marine Science Centre, Umeå University, 905 71 Hörnefors, Sweden; Department of Occupational Medicine, Umeå University, 901 87 Umeå, Sweden

**Keywords:** Climate change, Allochthonous organic matter, Primary production, Bacterial production, Food web, Monitoring

## Abstract

Climate change is likely to have large effects on the Baltic Sea ecosystem. Simulations indicate 2–4 °C warming and 50–80 % decrease in ice cover by 2100. Precipitation may increase ~30 % in the north, causing increased land runoff of allochthonous organic matter (AOM) and organic pollutants and decreased salinity. Coupled physical–biogeochemical models indicate that, in the south, bottom-water anoxia may spread, reducing cod recruitment and increasing sediment phosphorus release, thus promoting cyanobacterial blooms. In the north, heterotrophic bacteria will be favored by AOM, while phytoplankton production may be reduced. Extra trophic levels in the food web may increase energy losses and consequently reduce fish production. Future management of the Baltic Sea must consider the effects of climate change on the ecosystem dynamics and functions, as well as the effects of anthropogenic nutrient and pollutant load. Monitoring should have a holistic approach, encompassing both autotrophic (phytoplankton) and heterotrophic (e.g., bacterial) processes.

**Electronic supplementary material:**

The online version of this article (doi:10.1007/s13280-015-0654-8) contains supplementary material, which is available to authorized users.

## Introduction

The Baltic Sea is exposed to many stressors, e.g., eutrophication, organic pollutants, overfishing, invasive species, and acidification. Of these disturbances, eutrophication is presently considered to have the most severe effects in the Baltic proper, while organic pollutants are the largest environmental problem in the Gulf of Bothnia. Climate change may worsen these problems and it is thus a challenge to try to understand how different basins of the Baltic Sea may be influenced and how to appropriately manage this vulnerable ecosystem.

Climate change, induced by anthropogenic emissions of greenhouse gasses, is expected to have a significant impact on the Baltic Sea (BACC I author team 2008; BACC II author team 2015). A warming trend is already evident in the Baltic region and will continue through the twenty first century. Due to the large variability of the climate system, only temperature and directly related variables, such as ice conditions, are likely to show statistically significant changes in the next few decades, whereas significant effects in the water cycle can only be expected later in the century.

The impact of climate change on the Baltic Sea environment can be estimated with the help of coupled physical–biogeochemical models in conjunction with downscaling techniques that link projected global climate change to regional scales (e.g., Meier et al. 2011a). However, Baltic Sea ecosystem projections suffer from the biases of global and regional climate models, uncertainty in greenhouse gas emissions, nutrient load scenarios, and ecosystem responses, as well as natural climate variability. Hence, ensemble simulations are essential to estimate uncertainties in the projections (e.g., Meier et al. 2006, 2011a).

When assessing the effects of climate change on marine ecosystems, it is important to understand the driving mechanisms for biological and chemical processes. These drivers can influence the marine ecosystem in a variety of ways, altering food web community structure, overall productivity, and the transport of pollutants. Such changes are of great significance for the health and sustainability of the marine ecosystem. In this study, we have therefore combined information from field and experimental studies with modeling simulations, to try to understand the effects of climate change on the Baltic Sea ecosystems.

## Physical changes

Model simulations indicate that the surface water layer will warm more than the deep water in all sub-basins (Meier et al. 2012b). Sea surface temperature (SST) changes are projected to be the largest in the Bothnian Bay and the Bothnian Sea during summer and in the Gulf of Finland during spring. According to Meier et al. (2012a), the mean summer SST will increase by about 2 °C in the southern and 4 °C in the northern Baltic Sea by the end of this century. The larger warming in the north is caused at least partly by the ice-albedo feedback (Meier et al. 2011b). Under a more optimistic scenario, the average SST may increase by only 1 °C (Neumann 2010).

The future reduction of the ice cover depends mainly on projected air temperature changes over the Baltic Sea in winter, whereas the other drivers, like wind speed, are less important (e.g., Meier et al. 2004). Despite substantial uncertainties, all available scenario simulations indicate a 50–80 % decrease in sea ice extent by 2100 (Meier 2006). This increase in open water conditions will influence winds, wave climate, and underwater light conditions (Eilola et al. 2013). Wave height is projected to increase in spring in large parts of the Gulf of Finland, the Bothnian Sea, and the Bothnian Bay; and mean spring irradiance may increase in previously ice-covered areas. Significantly increased well-mixed layer depths are expected in most of the Bothnian Bay and the Gulf of Finland.

**Fig. 1:**
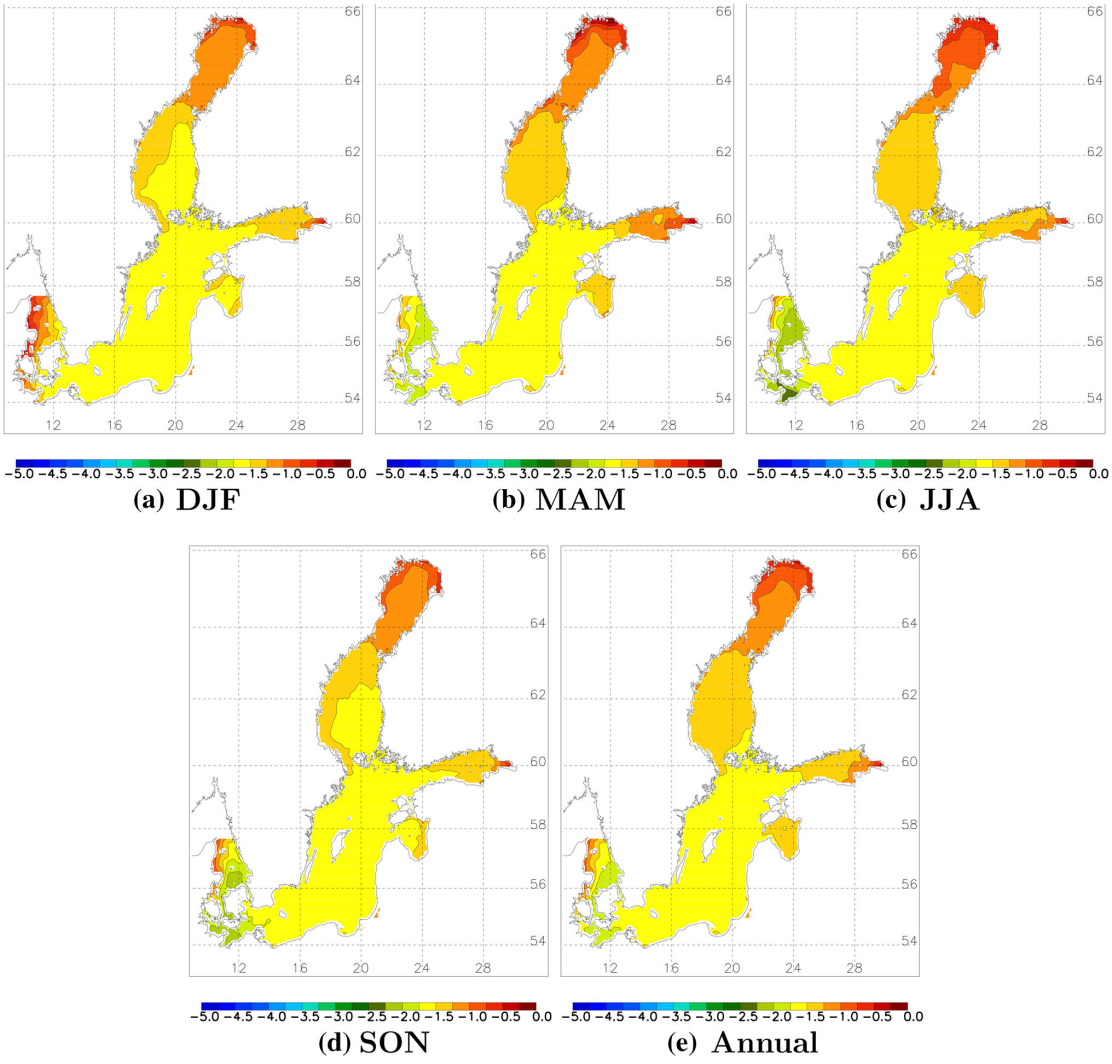
Projected seasonal (DJF = December to February, MAM = March to May, JJA = June to August, SON = September to November) and annual mean ensemble average sea surface salinity changes (in g kg^−1^) from 1978–2007 to 2069–2098 (from Meier et al. 2012a)

Sea surface salinity (SSS) will change less in the northern and eastern Baltic Sea (least in the Bothnian Bay) and most in the Danish straits region, especially the Belt Sea (Fig. 1) (Meier et al. 2012a), through shifting salinity fronts in the transition zone. The SSS changes are rather uniform across the seasons. In the ensemble mean, salinity in the strongly stratified Bornholm and Gotland basins decreases by 1.5–2 salinity units at all depths (Meier et al. 2012b). In the less strongly stratified Gulf of Finland and Bothnian Bay, salinity changes are larger in the deep water, reducing the vertical stability. These results are rather consistent among various Baltic Sea models. The largest, model-related uncertainty was found for projected halocline depth in the Baltic proper (Meier et al. 2012b).

Salinity changes (Fig. 1) are caused by changes in runoff, which in the depicted scenario simulations is projected to increase by 15–22 % (Meier et al. 2012a). Although all scenario simulations suggest either unchanged or decreased salinity compared to present climate (Meier et al. 2006), projections have large uncertainties due to variability among regional Baltic climate models in water balance (e.g., Kjellström and Lind 2009), wind projections (Kjellström et al. 2011; Nikulin et al. 2011), runoff projections using various hydrological models, and also bias in correction methods for air temperature and precipitation (Meier et al. 2012b; Donnelly et al. 2014).

Overall stratification changes in the Baltic proper are expected to be small because the greater freshwater supply will increase recirculation of brackish surface waters and consequently reduce saltwater influx to the Baltic Sea (Meier 2005). Changes in wind-induced mixing are more important for stratification changes in the Baltic proper than changes in runoff (Meier 2005).

Saltwater inflows from the Kattegat influence average salinity, vertical stratification, and deep water oxygen conditions in the Baltic Sea (e.g., Meier and Kauker 2003). Still Gräwe et al. (2013) found no clear tendency for salt water transport to change in the future climate, either during medium or major inflow events. Schimanke et al. (2014) estimated that atmospheric events favorable for major Baltic inflows may become slightly more common. All published scenario simulations share the weakness that they have not considered the effect of global sea-level rise on saltwater inflows. Unpublished research by H.E.M. Meier et al. indicates that if the global mean sea level rises by 1.5 m, then the effect on salinity and stratification cannot be neglected.

## Pelagic food web structure from north to south

To evaluate the effects of climate change on the marine food web, it is crucial to understand the mechanisms regulating food web structure and productivity. Bacteria and phytoplankton are key organisms at the base of the marine food web, providing biomass production on which the rest of the food web relies. They take up nutrients via diffusion and are thus the first organisms to respond to changes in nutrient availability. Their interaction strongly influences the structure and efficiency of the food web and hence production at higher trophic levels.

Phytoplankton primary production increases strongly basin by basin from north to south in Baltic Sea offshore waters and is almost tenfold higher in the Baltic proper than in the Bothnian Bay (Fig. S1, Supplementary Material). The low primary production in the Bothnian Bay is caused by a combination of strong phosphorus (P) limitation (Andersson et al. 1996), a short productive season and a poor light climate for phytoplankton. The Bothnian Bay water is relatively brown, with twice the concentration of humic substances and ~70 % lower phosphorus concentration compared to the Baltic proper (Fig. S1).

The annual bacterial biomass production, on the other hand, is rather uniform from north to south, although its relative share of the total production varies among the basins. In offshore waters of the Bothnian Bay, the Bothnian Sea, and the Baltic proper, the bacterial production equals 70, 30, and 12 % of the primary production, respectively (Fig. S1). The large importance of bacteria in the north may partly be due to high concentrations of allochthonous organic matter (AOM), e.g., humic substances, which fuel bacteria with external organic matter (Sandberg et al. 2004). Another contributing factor can be low nutrient availability (P), as phytoplankton might exude more of their photosynthetic products when nutrients are scarce. These photosynthetic exudates are then channeled into bacterial production.

## Drivers of coastal production

Field studies were performed during the spring period in three estuaries in the Baltic Sea, in order to find out how the river inflow influences primary and bacterial production. In the Öre estuary, Gulf of Bothnia, primary production was stimulated by increased phosphorus availability and hampered by high concentration of dissolved organic carbon (DOC) (Table S1). DOC, humic substances, and colored dissolved organic matter (CDOM) are all measures of allochthonous organic matter, which is transported from land to the Baltic Sea via rivers, reducing light penetration in coastal waters (Pettersson et al. 1997). In the nitrogen-limited Emån estuary, in the southern Baltic Sea, the spring primary production was negatively related to phosphorus and temperature (Table S1).

Bacterial production was mainly controlled by factors that are likely to be influenced by climate change. A stimulation by humic substances and DOC was observed in the coastal areas of the Gulf of Bothnia (Råne and Öre estuaries) (Table S1). Even though the bioavailable fraction of the AOM in short-term experiments is small (Stepanauskas et al. 2002; Lignell et al. 2008), the large AOM export via rivers to the coastal zone, combined with increased bioavailability by, e.g., photochemical processes over longer time scales, makes AOM a major driver of bacterial production in the northern Baltic Sea (Sandberg et al. 2004). In the study area in the southern Baltic Sea, however, regression analysis indicated temperature as the major factor influencing bacterial production (Table S1). This agrees with previous studies in the Baltic Sea, indicating that bacterial production is temperature limited below +6 to +8 °C, but substrate limited (by inorganic nutrients or organic C) at higher temperatures (e.g., Autio 1998).

## Effects of increased dissolved organic matter inflow on coastal production

An integrated understanding of the effects of the projected increase in runoff in the northern Baltic Sea is required. The fertilizing effect of nitrogen and phosphorus discharged by rivers has been the main issue in recent research (Smith 2006; Finkel et al. 2010). Nutrient-rich freshwater discharge can lead to eutrophication, with increased phytoplankton growth, oxygen consumption, hypoxia, and reduced recreational value of the coastal zone. However, freshwater discharge also has other potential effects on the hydrography, chemistry, and biology of the marine environment. Dissolved organic matter (DOM) is a major chemical constituent of river water, with potential effects in coastal areas. Colored humic substances degrade the light climate, heat the near-surface water (Cole et al. 1992; Howarth et al. 2000; Ask et al. 2009), and can also act as a carbon source for bacterioplankton production (Wikner et al. 1999).

Using a 13-year ecological time-series from the Bothnian Bay, the Bothnian Sea, and the Öre estuary, Wikner and Andersson (2012) studied the effect on the trophic balance of a 4-year period (1998–2001) with elevated river discharge. The ratio between phytoplankton and bacterial production was used as an index of trophic balance. Correlation analysis indicated that increased freshwater discharge of colored DOM reduced the primary production, while bacterial production remained stable. Previous studies have shown that part of the riverine DOM can be assimilated by bacterioplankton (Zweifel et al. 1995; Wikner et al. 1999). This suggested a dual effect of riverine DOM on the trophic balance, by reducing light (i.e., energy) supply to photosynthetic organisms, and simultaneously stimulating heterotrophic organisms by providing an alternative carbon and energy source. It is likely that the food web efficiency decreased during this period, since bacteria-based food webs generally have more trophic levels than those based on phytoplankton (Berglund et al. 2007). However, in a climate change experiment combining increased input of humic substances with higher temperature, the food web efficiency was not reduced when the planktivorous fish was the three-spine stickleback (*Gasterosteus aculeatus*), which is able to adapt to high temperature and can exploit the system efficiently (Lefébure et al. 2013).

Benthic zones are often important contributors to overall production in shallow coastal waters. During the 1998–2001 period of elevated river flow and lower pelagic primary production, the native benthic amphipod *Monoporeia affinis* declined drastically in the Gulf of Bothnia (Eriksson-Wiklund and Andersson 2014), probably due to food shortage, since settling phytoplankton is its main food source. In the virtual absence of this amphipod, the non-native polychaete *Marenzelleria* spp. invaded the area. Such changes in species composition alter food web structure and resource use and can modify the transport and release of pollutants.

## Effects of increased DOC inflow on organic pollutant sorption and bioaccumulation

The seasonal export of hydrophobic organic contaminants from soils to river water peaks during the spring flood, and is connected to processes determining DOC release at the soil–water interface (Bergknut et al. 2010). Climate-induced increases in precipitation and DOC release are therefore likely to cause increased inflow of contaminants to coastal areas.

**Fig. 2:**
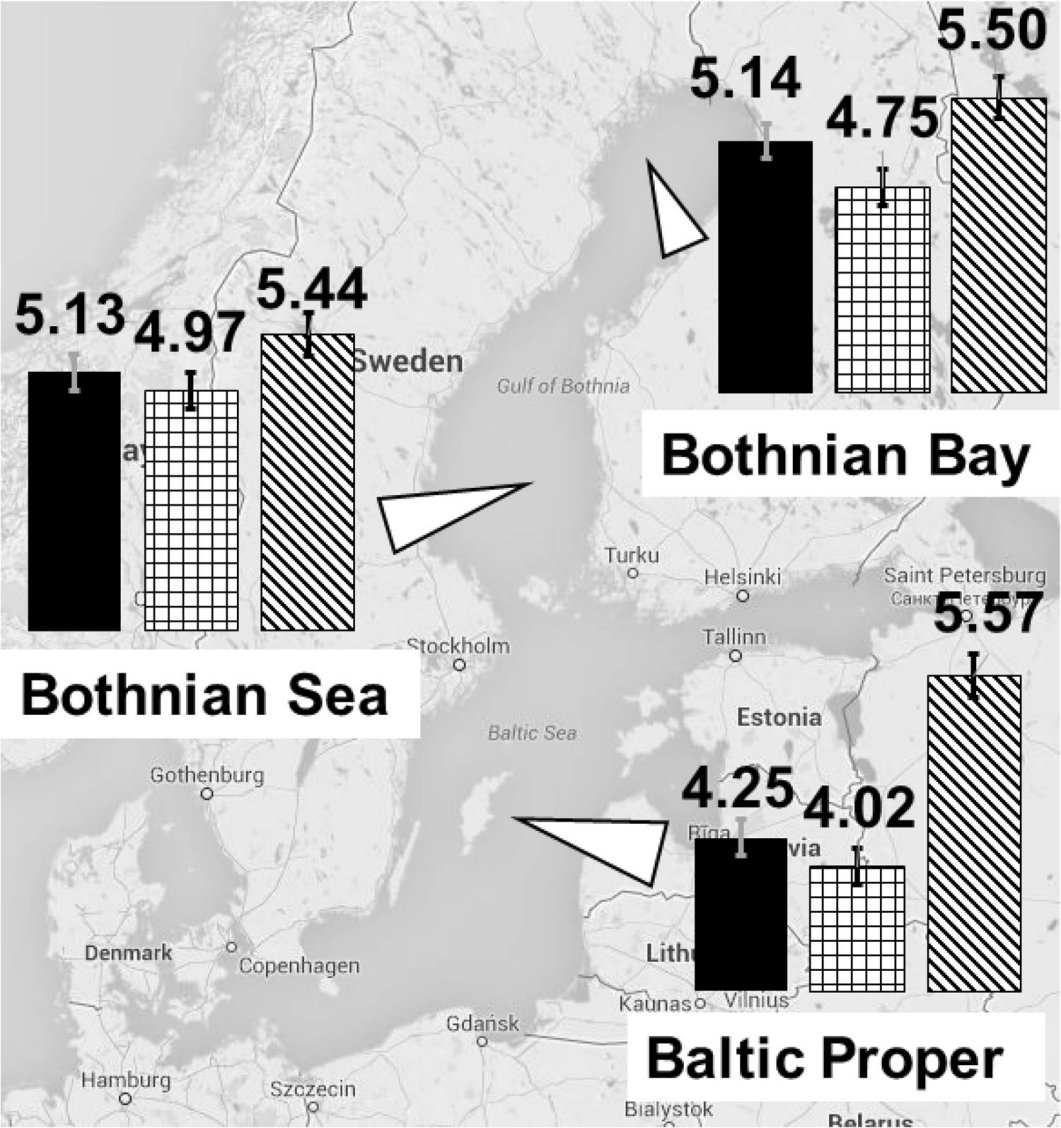
Log KDOC values for hexachlorobenzene (*black*), phenanthrene (*squared*), and tributyl phosphate (*striped)* at different locations in the Baltic Sea. Water samples were collected along a north–south gradient in August 2013, filtered to retrieve the dissolved fraction, and spiked with different organic pollutants. *Error bars* represent the standard deviation of data from four sampling points in each basin

Higher concentrations of DOC in the seawater may also affect the fate of the pollutants, e.g., by changing the effects on their solubility, volatilization, long-range transport, transformations, and bioavailability (e.g., Kukkonen et al. 1990; Poerschmann and Kopinke 2001). In a spiking experiment, we demonstrated that the sorption of structurally diverse pollutants to dissolved organic matter in seawater varies between areas of the Baltic Sea (Fig. 2) (Ripszam et al. 2015). The distribution constant between water and DOC (log K_DOC_) for hexachlorobenzene and phenanthrene, representing halogenated aromatic and polycyclic aromatic compounds, respectively, decreased from north to south in the Baltic Sea, while tributyl phosphate, representing compounds with polar functional groups and relatively high water solubility, showed no geographical variation. The salinity gradient in the study, from 2.8 to 6.6, probably had only a minor effect on the partitioning of the tested organic pollutants (Engebretson and von Wandruszka 1994; Kuivikko et al. 2010). Considering the small variation of DOC concentration in the offshore Baltic Sea (4.2–5.2 mg C/l), we propose that differences in quality, i.e., the terrestrial component of the DOC, caused the differences in log K_DOC_ values.

## Scenarios for ecosystem changes

The combined influence of changing climate, eutrophication, acidification, and overfishing on the marine ecosystem has been studied with coupled physical–biogeochemical–carbonate models (e.g., Neumann 2010; Meier et al. 2011a, b; Neumann et al. 2012; Omstedt et al. 2012) and food web models (e.g., Niiranen et al. 2013). However, models coupling lower and higher trophic levels do not exist, and our knowledge of the effects of changing climate and other anthropogenic drivers on the marine ecosystem is still very limited. Eilola et al. (2011) compared three physical–biogeochemical Baltic Sea models and identified four major sources of uncertainty: (1) uncertain initial conditions, (2) unknown bioavailability of nutrients in land runoff, (3) unreliable parameterization of sediment fluxes and turnover of nutrients in the sediments, and (4) lack of process understanding in the Gulf of Bothnia. In addition, the possible influence of genetic adaptation is currently unpredictable. Nevertheless, projected future hydrographic conditions such as mixing depth, light penetration, vertical exchange of nutrients and oxygen, and deep water ventilation will very likely affect biogeochemical cycles and consequently also the entire ecosystem. Given the projected changes in the abiotic environment and biogeochemical processes, we cautiously suggest some likely future changes in the marine ecosystem.

**Fig. 3:**
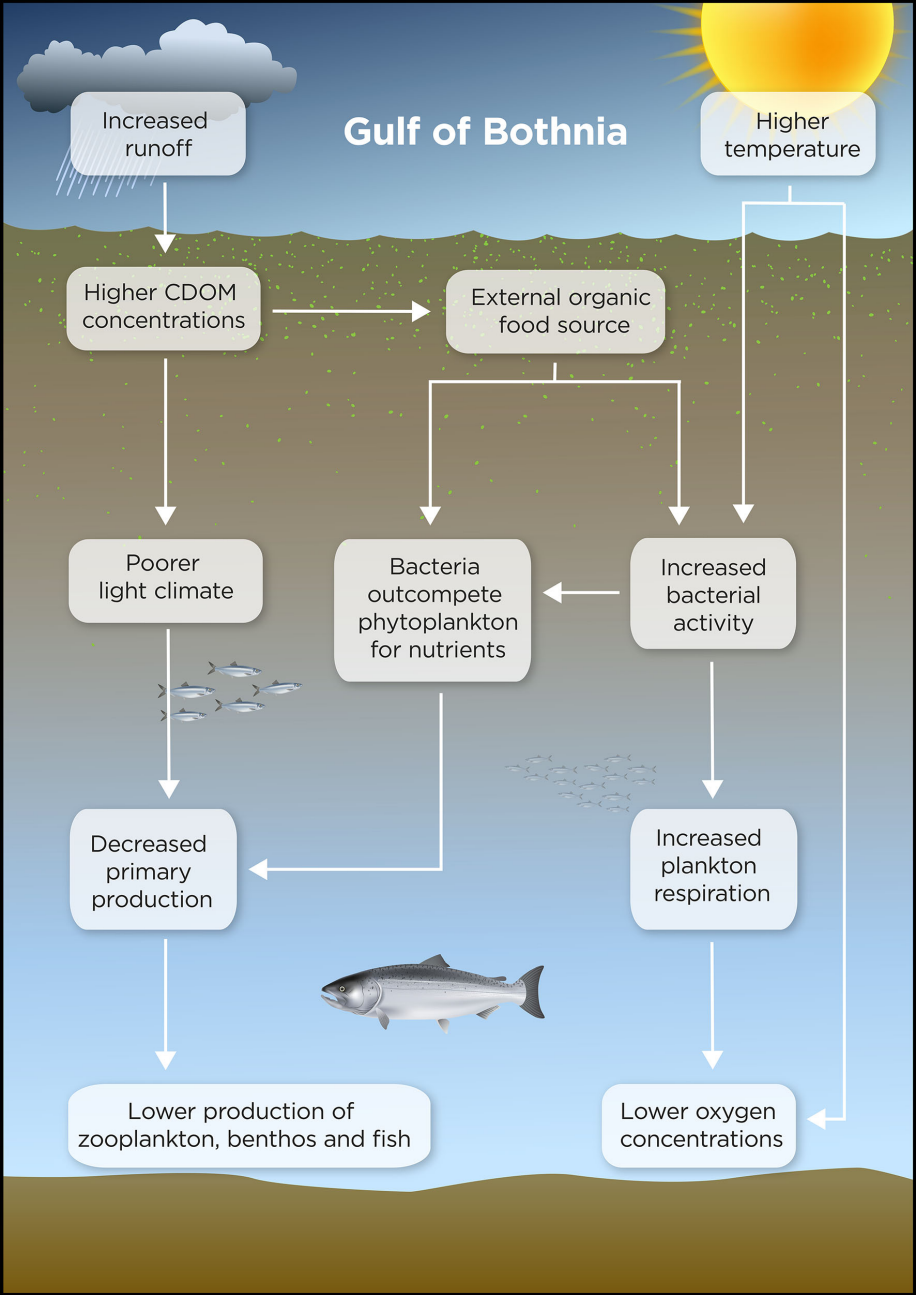
Conceptual model of climate-induced ecosystem changes in the Gulf of Bothnia by 2100. Illustration: Kristina Viklund and Mattias Pettersson

Ice cover is expected to decrease in the northern Baltic Sea, causing an earlier onset of the spring bloom by up to 1 month (Fig. 3), and increased wind- and wave-induced resuspension will hasten the transport of nutrients from the coastal zone to the open sea (Eilola et al. 2013). Increased river runoff will lead to higher concentrations of AOM, which reduces light penetration in the water and potentially also primary production (Wikner and Andersson 2012; Lefébure et al. 2013). The AOM provides an alternate carbon source for heterotrophic bacteria as compared to phytoplankton-derived substrates, which may increase bacterial activity, making bacteria outcompete phytoplankton for inorganic nutrients. The poorer light climate and increased competition from bacteria may decrease phytoplankton production (Lefébure et al. 2013). Although the spring bloom occurs earlier due to earlier ice break-up, primary production does not necessarily increase according to modeling results (Eilola et al. 2013). Should the yearly primary production decrease, it is likely that the production will decrease also at higher trophic levels. The food web will get an additional intermediate trophic level, which will cause increased respiration and excretion losses (Fig. 5), most likely causing decreased production of zooplankton, benthos, and fish. Because water absorbs less oxygen at higher temperature, oxygen concentration in the water will be reduced, and increased AOM will stimulate bacterial respiration, further reducing the oxygen concentration (Panigrahi et al. 2013). Decreased light availability may favor tactile over visual predators. Taken together, field observations in the coastal zone (data presented here), experimental studies (e.g., Lefébure et al. 2013), and long-term, large-scale studies (Wikner and Andersson 2012) indicate that the base of the food web, i.e., the phytoplankton and bacteria, can become significantly altered if climate change increases inputs of AOM to the Gulf of Bothnia.

**Fig. 4:**
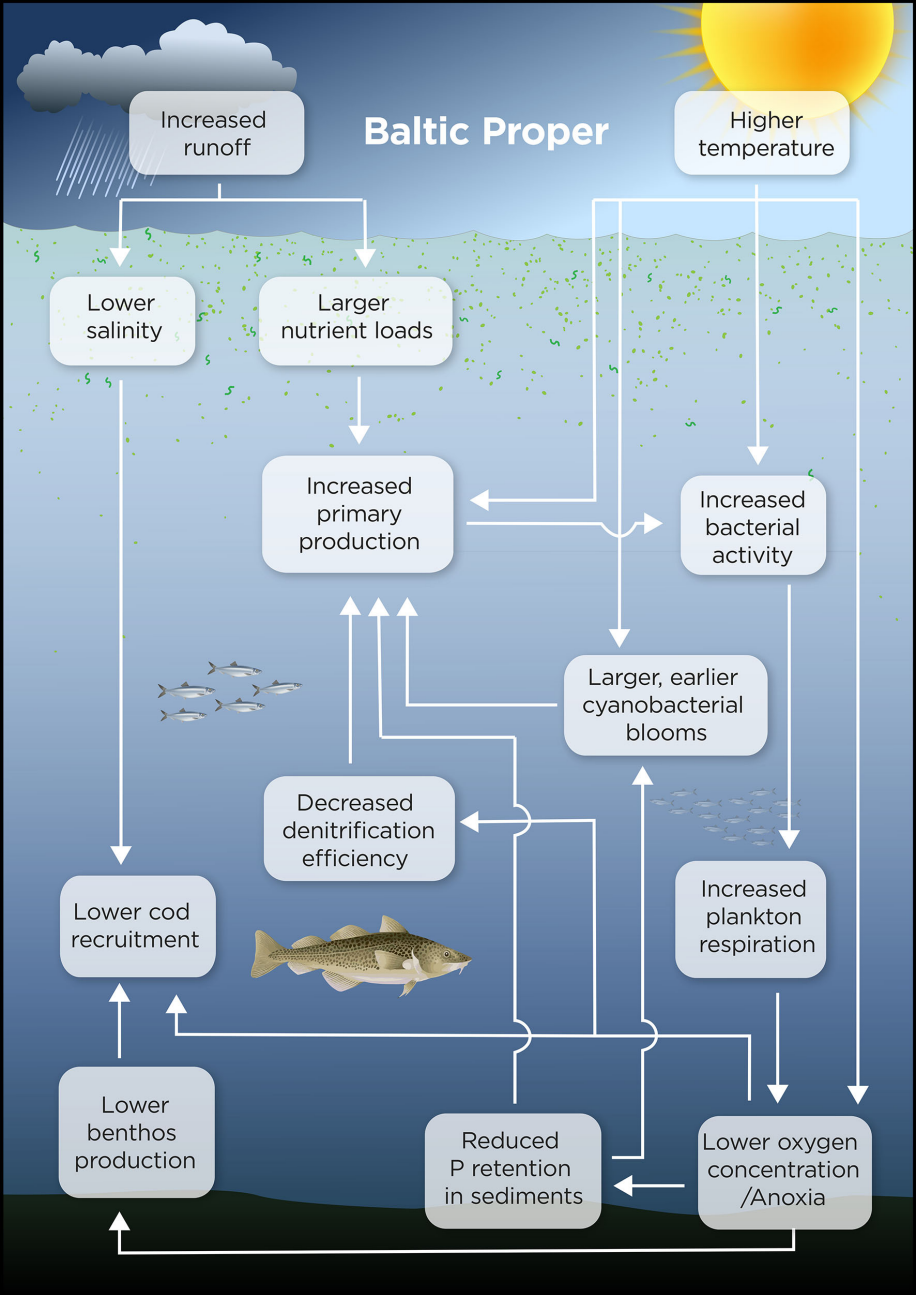
Conceptual model of climate-induced ecosystem changes in the Baltic Proper by 2100. Illustration: Kristina Viklund and Mattias Pettersson

**Fig. 5:**
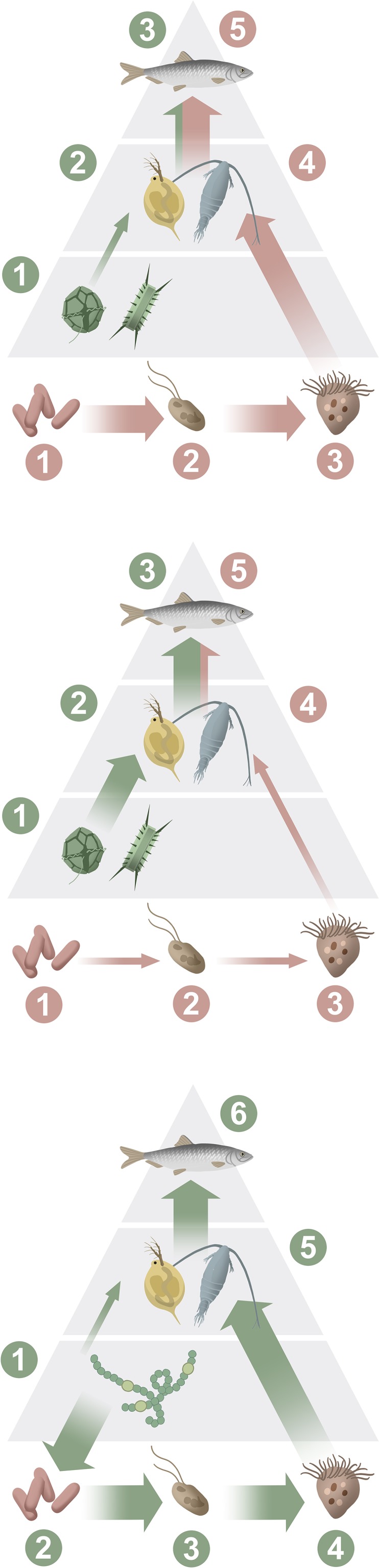
Simplified schematic view of climate-altered food webs in the Bothnian Bay (*upper*), southern Bothnian Sea (*mid*), and Baltic proper (*lower*) in summer. *Green arrows* represent autochthonous and *brown arrows* allochthonous production. Organisms included in the food webs and their trophic position (in parenthesis); bacteria (*1*,* 2*), phytoplankton (*1*), flagellates (*2*,* 3*), ciliates (*3*,* 4*), zooplankton (*2*,* 4*,* 5*), and fish (*3*,* 5*,* 6*). Illustration by Mats Minnhagen

In the Baltic proper, increased nutrient loads and higher temperatures may intensify internal nutrient cycling (Meier et al. 2011a), potentially increasing both primary production and oxygen consumption (Fig. 4). This may possibly increase phosphorus mobility and reduce denitrification efficiency (Meier et al. 2012b). Without drastic nutrient load abatements, hypoxic and anoxic areas are projected to increase (Meier et al. 2011a) and cause intensified exchange of nutrients between shallow and deeper waters (Eilola et al. 2012).

Scenario simulations suggest that the rising atmospheric CO_2_ will control future pH changes in the surface water and that eutrophication will not affect the mean pH (Omstedt et al. 2012). Climate warming may lead to earlier and more frequent cyanobacterial blooms, as already observed for surface accumulations of cyanobacteria (Kahru and Elmgren 2014) and perhaps also to increased nitrogen fixation (Meier et al. 2012b; Neumann et al. 2012; Hense et al. 2013). Nitrogen-fixing cyanobacteria will supply the ecosystem with plant-available nitrogen, but, as they are of poor food quality for consumers, the efficiency of energy transfer to higher trophic levels may be reduced (Fig. 5).

A lower salinity may reduce or eliminate cod spawning areas, reducing the value of the fish catch (Fig. 4). Anoxia will reduce the production of benthic fish food. A lower salinity will eliminate many marine species, but allow more freshwater species to colonize the ecosystem. A decrease in salinity will inevitably change species composition and therefore ecosystem function (e.g., through the loss of filter-feeding bivalves). Most food-web and fish population models indicate that cod fishing will remain an important determinant of the cod stock in the future, although climate effects may be substantial. Eutrophication has, however, been suggested to be of minor importance for the cod stock size (Niiranen et al. 2013).

Climate change may directly benefit invasive species by providing conditions nearer to those in their native ranges, e.g., warmer temperatures. Furthermore, invasive species tolerant of low-oxygen conditions may be favored, such as *Marenzelleria* spp. (Maximov 2011). As these polychaetes burrow deep into sediments (30 cm), they can stimulate release and subsequent bioaccumulation of buried organic pollutants (Josefsson et al. 2010, 2011).

In a future climate, halogenated aromatic and polycyclic aromatic pollutants may be sorbed to DOC to a greater extent. Whether this will increase or reduce their availability to marine organisms is unknown. Since DOC is partly utilized by bacteria as a food source, we hypothesize an increased transport of DOC-associated pollutants up the food web via microbes (Wallberg et al. 2001). On the other hand, high concentrations of humic substances in lakes have been shown to make persistent organic pollutants less available for uptake by fish (Larsson et al. 1992).

## Implications for management of the Baltic Sea

Considering the complex and interactive alterations climate change may induce in the Baltic Sea ecosystem, it is crucial that future Baltic Sea management takes these aspects into consideration. During recent decades, the Baltic countries have agreed on joint goals for the management of the Baltic Sea environment. Compliance monitoring has been used to classify the ecological state of marine waters (e.g., Anon 2007), but is not yet focused on climate change, which interacts with other ecosystem changes and exposures.

One example is chlorophyll *a*, a proxy for phytoplankton biomass, which should respond significantly to nutrient loading. The chlorophyll content in algae is, however, highly variable depending on, e.g., species composition and local light climate, which in turn are affected by AOM in the water. An increased AOM load is likely to promote bacterial production, but decrease primary production, as described above. The oxygen concentration may decrease and the ecosystem may become more heterotrophic. The food web will acquire extra trophic levels, leading to greater energy losses and decreased fish production. We therefore suggest that not only primary production but also bacterial production should be monitored in the Baltic Sea.

Management should also consider the effects of temperature and DOC on hypoxia, and thus not only interpret low oxygen levels as a result of enrichment with nitrogen and phosphorus. We found markedly higher temperature sensitivity of bacterioplankton in coastal environments with high concentrations of DOC. Thus, climate-related factors (i.e., temperature and runoff) can influence hypoxia in estuarine waters, and in such cases reduction of nitrogen and phosphorus alone may not be an effective restoration measure.

Climate-induced changes in geochemical and biological conditions will alter the concentration and distribution of semi-persistent and persistent organic pollutants in the sea. The net effect is expected to differ among pollutants depending on their lipophilicity, sorption properties, volatility, and resistance to metabolism. This is important to consider when forecasting food web transfer and human exposure and when designing or modifying monitoring programs for such pollutants (Undeman et al. 2015).

It has been suggested that, because the invasive deep-burrowing polychaete *Marenzelleria* stimulates the release of legacy pollutants from fiber banks and other hotspots in the Gulf of Bothnia (Josefsson et al. 2010, 2011), it may have played a part in a recent local reproduction failure in white-tailed eagle (*Haliaeetus albicilla*) (Helander and Bignert 2012). Although the interactions between native food webs, invasive species, pollutant chemistry, and climate change are complex, they clearly need to be considered when managing marine ecosystem.

While a number of management plans have been developed in response to eutrophication (e.g., the HELCOM Baltic Sea Action Plan, http://helcom.fi/baltic-sea-action-plan and the EU water framework directive), monitoring needs to be expanded also to cover secondary producers and other processes and to encompass changes in climate and anthropogenic pollutant loads.

## Electronic supplementary material

Supplementary material 1 (PDF 97 kb)
