# Supplementary material for: Projected future climate change and Baltic Sea ecosystem management
Source: Ambio. 2015 May 28;44(Suppl 3):345–56. doi: 10.1007/s13280-015-0654-8 (PMC4447695; doi:10.1007/s13280-015-0654-8)
Supplement: Supplementary file 1 — Supplementary material 1 (PDF 97 kb) [file 13280_2015_654_MOESM1_ESM.pdf]

AMBIO
Electronic Supplementary Material

This supplementary material has not been copy edited by the publisher or the editorial office.

Title: **Projected future climate change and Baltic Sea ecosystem management**

Authors: Agneta Andersson, H.E. Markus Meier; Matyas Ripszam, Owen Rowe, Johan Wikner, Peter Haglund, Kari Eilola, Catherine Legrand, Daniela Figueroa, Joanna Paczkowska, Elin Lindehoff, Mats Tysklind, Ragnar Elmgren

Table S1 Analysis of the relationship of primary production* and bacterial production** to potential explanatory factors in the Råne (Bothnian Bay), Öre (Bothnian Sea) and Emån (Baltic proper) estuaries in spring, with stepwise linear multiple regression*** (IBM SPSS statistics, version 21).

Estuary	Variable	Mod.r^2	Model Sign.	Factor	Beta/Slope	Factor sign.
**Råne**	Prim Prod	-				
	Bact Prod	0.68	<0.001	Humics	+0.83	<0.001
**Öre**	Prim Prod	0.51	<0.001	Tot P	+0.59	<0.001
				DOC	-0.45	<0.001
				Temp	+0.24	0.023
	Bact Prod	0.50	<0.001	Temp	+0.65	<0.001
				DOC	+0.28	0.010
**Emån**	Prim Prod	0.61	<0.001	P	-1.20	<0.001
				Temp	-0.78	0.001
	Bact Prod	0.62	<0.001	Temp	+0.79	<0.001

* Factors tested for primary production: temperature, P, N, Secchi depth, humic substances, DOC, colored dissolved organic matter (CDOM), SPM and bacterial production.

** Factors tested for bacterial production: temperature, P, N, humic substances, DOC, CDOM, suspended particulate matter (SPM) and primary production.

*** Data were ln-transformed to obtain normal distributions. Linearity of the data were checked using Q-Q plots. Models with significant predictors are shown per estuary and

dependent variable. There was no correlation between important explanatory factors that hampered the step-wise multiple regression. Surface water was sampled in the Råne estuary (18 stations, sampled 2 times, May-June), Öre estuary (18 stations, sampled 3 times, May-early June) and the Emån estuary (8 stations, sampled 4 times, April-May).

Primary production was measured *in situ* using the ^{14}C technique, bacterial production using the 3HT thymidine (Råne and Öre estuaries) and 3HT leucine (Emån estuary) techniques. Physicochemical measurements used standard techniques (HELCOM Combine 2014).

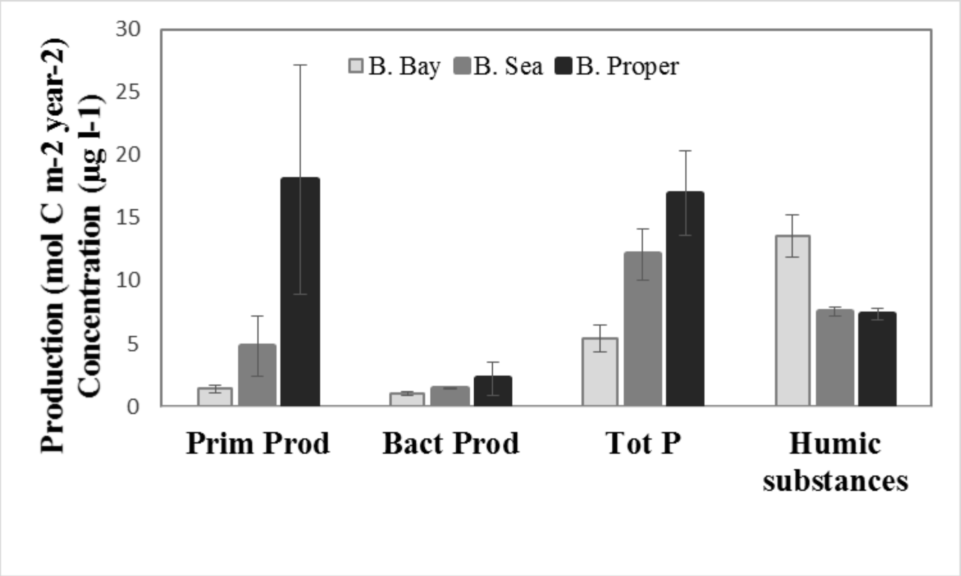

Fig. S1 Annual primary and bacterial production, and average total phosphorus and humic substance concentrations in the photic zone in the Bothnian Bay (B. Bay), Bothnian Sea (B. Sea) and the Baltic proper (B. proper). Production recalculated from Samuelsson et al. (2006), Larsson et al. (2010) and Legrand (2015, this issue). Tot P and humic substances were measured at 13 stations along a north-south gradient in August 2011 and March 2012 using HELCOM standard methods. Error bars are standard deviation.

References

HELCOM Combine 2014. A manual for marine monitoring in the combine program of HELCOM http://helcom.fi/Documents/Action%20areas/Monitoring%20and%20assessment/Manuals%20and%20Guidelines/Manual%20for%20Marine%20Monitoring%20in%20the%20COMBINE%20Programme%20of%20HELCOM.pdf

Larsson, U., S. Nyberg, K. Andreasson, O. Lindahl, and J. Wikner. 2010. Phytoplankton production – measurements with problems. *Havet 2010,* 26-29. Havsmiljöinstitutet (in Swedish)

Legrand, C., E. Fridolfsson, M. Bertos-Fortis, E. Lindehoff, P. Larsson, J. Pinhassi, and A. Andersson 2015. Microbial production in coastal and offshore food webs of the changing Baltic Sea. *AMBIO* this issue

Samuelsson, K., J. Berglund, and A. Andersson 2006. Factors structuring the heterotrophic nanoflagellate and ciliate community along a brackish water productivity gradient. *Journal of Plankton Research* 28: 345–359.
